# LncRNAs in the *Dlk1-Dio3* Domain Are Essential for Mid-Embryonic Heart Development

**DOI:** 10.3390/ijms25158184

**Published:** 2024-07-26

**Authors:** Xiangqi Teng, Hongjuan He, Haoran Yu, Ximeijia Zhang, Jie Xing, Jiwei Shen, Chenghao Li, Mengyun Wang, Lan Shao, Ziwen Wang, Haopeng Yang, Yan Zhang, Qiong Wu

**Affiliations:** 1 Faculty of Life Sciences and Medicine, School of Life Science and Technology, Harbin Institute of Technology, Harbin 150001, China; ethel555@163.com (X.T.); hehongjuan0727@163.com (H.H.); yhr0823@sina.com (H.Y.); zhangximeijia95@hotmail.com (X.Z.); xjaixuexi@163.com (J.X.); sjw_33113@163.com (J.S.); lichenghao.bioinformatics@gmail.com (C.L.); mengyunwang@yeah.net (M.W.); orchidshao7@gmail.com (L.S.); hitwangziwen@163.com (Z.W.); yanghp11@126.com (H.Y.); zhangtyo@hit.edu.cn (Y.Z.); 2 State Key Laboratory of Urban Water Resource and Environment, Harbin Institute of Technology, Harbin 150001, China

**Keywords:** *Dlk1-Dio3* domain, cardiac development, epicardial cell, proliferation

## Abstract

The *Dlk1-Dio3* domain is important for normal embryonic growth and development. The heart is the earliest developing and functioning organ of the embryo. In this study, we constructed a transcriptional termination model by inserting termination sequences and clarified that the lack of long non-coding RNA (lncRNA) expression in the *Dlk1-Dio3* domain caused the death of maternal insertion mutant (MKI) and homozygous mutant (HOMO) mice starting from E13.5. Parental insertion mutants (PKI) can be born and grow normally. Macroscopically, dying MKI and HOMO embryos showed phenomena such as embryonic edema and reduced heart rate. Hematoxylin and eosin (H.E.) staining showed thinning of the myocardium in MKI and HOMO embryos. In situ hybridization (IHC) and quantitative reverse-transcription polymerase chain reaction (qRT-PCR) showed downregulation of lnc*Gtl2*, *Rian*, and *Mirg* expression in MKI and HOMO hearts. The results of single-cell RNA sequencing (scRNA-Seq) analysis indicated that the lack of lncRNA expression in the *Dlk1-Dio3* domain led to reduced proliferation of epicardial cells and may be an important cause of cardiac dysplasia. In conclusion, this study demonstrates that *Dlk1-Dio3* domain lncRNAs play an integral role in ventricular development.

## 1. Introduction

During development, the heart is the earliest organ to form and function, and its development is a complex and sophisticated process [1,2]. For mammalian vertebrates, four chambers, thickened ventricular walls, and a functional coronary vascular system are necessary for a morphologically mature heart [3]. A key morphogenetic process during heart development is myocardial compaction, which is necessary for the formation of functional ventricular walls [3]. In humans, congenital heart disease is the most common inherited birth defect, affecting about 1% of live births, and the most common cause of embryonic and fetal mortality [4,5]. Therefore, research on cardiac development is crucial. Early cardiac morphogenesis includes cardiac crescent and tube formation, followed by a process that ultimately leads to asymmetric rings of chamber formation [2]. In terms of the ventricular wall, the trabecular ridge begins to appear at embryonic E9.0, whereby the trabecular myocardium begins to form. At E9.5–13.5, the mature trabecular myocardium is formed and facilitates the exchange of oxygen and nutrients, and at E14.5, the trabecular myocardium gradually compacts to form dense myocardium, which fuels ventricular contraction. The heart consists of several different types of cells, including cardiomyocytes (CMs) (including atrial and ventricular cells) and non-CMs (such as endocardial, epicardial, smooth muscle cells, and others). CMs are specialized heart cells with contractile functions [6]. The endothelial cell (Endo) plays an important role in cardiac development and cardiac function as a major component of trabecular formation, vascular structure, and valves [7]. Smooth muscle cells (SMCs) are an important component of the cardiac vessel wall and provide major structural support for the cardiac outflow tract (OFT) [8].

The epicardium is the outermost layer of the vertebrate heart and is a conserved mesothelial cell that serves as a source of pluripotent progenitor cells during cardiac embryogenesis [9,10]. Studies have shown that the epicardium contributes to the formation of different cardiac cell lineages including pericytes (PCs), SMCs, and fibroblasts (Fibs) during cardiac development and has an important role in the formation of the coronary system and development of the ventricular wall [11,12]. Genetic or surgical ablation of the epicardium has been shown to lead to hypoplasia of the dense zone and the interruption of myocardial growth [13]. The epicardium is not only an epithelial layer encasing the heart but also an important source of cells, extracellular matrix (ECM) components, and paracrine factors required for the formation of dense myocardium and the pattern of the coronary vasculature system [14].

Genomic imprinting has an important role in embryonic development. Imprinted genes are a class of genes that depend on the asymmetric expression of parental origins, and their normal and ordered expression can regulate the process of fetal growth and development during pregnancy [15,16,17]. The *Dlk1-Dio3* domain is located at the end of human chromosome 14 and mouse chromosome 12 and includes the paternally expressed protein-coding genes *Dlk1*, *Rtl1*, and *Dio3* and the maternally expressed long non-coding RNAs (lncRNAs) *Gtl2*, *Rian*, *Mirg*, and a large number of microRNAs (miRNAs) [18]. Most maternally expressed non-coding RNAs in the *Dlk1-Dio3* domain are transcribed by a large polycistronic transcription unit regulated by the *Gtl2* promoter [18,19]. Several studies have reported that lncRNAs within this region are important for the development of heart disease and Endo function [20,21]. A gene ablation study is a standard research tool for evaluating the function of coding proteins or non-coding RNAs. To date, three reports of *Dlk1-Dio3* domain knockout mouse models are available, and distinct phenotypes were observed in different model mice when the knockout location or length was different [22,23,24]. Moreover, changes in heart development were not focused on in any of these studies.

To investigate the effect of lncRNAs in the *Dlk1-Gtl2* domain on mouse embryonic heart development, we constructed a mouse model of transcription termination. Here, our data showed that defective expression of lncRNAs leads to embryonic death starting at E13.5 and results in hypoplasia of the ventricular wall of the embryonic heart. In parallel, we used single-cell RNA sequencing (scRNA-seq) to analyze the effects of lncRNAs in the *Dlk1-Dio3* domain in cardiac development, especially on epicardial cells (Epis). Based on these observations, this study aimed to show that lncRNAs in the *Dlk1-Dio3* domain play a key role in mouse embryonic heart development, with hopes that insights into the role of the *Dlk1-Dio3* domain in cardiac development may provide new information.

## 2. Results

### 2.1. Construction of a Terminating Sequence Insertion Model

**Figure 1:**
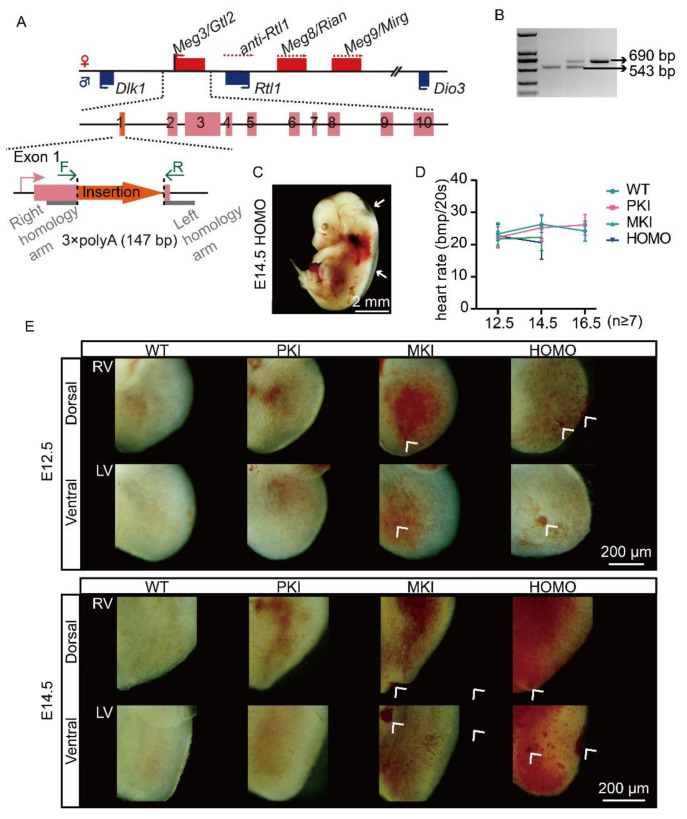
Schematic of model construction and the growth phenotypes of the mutants. (**A**) Schematic representation of the *Dlk1-Dio3* domain at the end of mouse chromosome 12 and the location of transcription termination sequence insertion. Maternally expressed genes are indicated by red, paternally expressed genes by blue, the 10 exons of *Gtl2* are indicated in pink, the inserted sequence is in orange, flanked by gray rectangles for the left and right homology arms, respectively. The positions of the genotyping primers are indicated by green arrows. (**B**) Genotyping of embryos. Knock-in band length 690 bp, WT band 543 bp. (**C**) HOMO mutant embryos of E14.5 with edema (white arrows point to edema). Scale bars, 2 mm. (**D**) Heart rate statistics of E12.5, 14.5, 16.5 embryos (E12.5: WT n = 13, PKI n = 9, MKI n = 7, HOMO n = 10; E14.5: WT n = 32, PKI n = 20, MKI n = 15, HOMO n = 13; E16.5: WT n = 10, PKI n = 9). Data are expressed as the mean ± SD. (**E**) Dorsal-ventral view of E12.5 and E14.5 embryonic heart (white arrows point to areas of abnormal heart development). Scale bars, 200 μm. RV, right ventricle; LV, left ventricle.

To terminate the transcription of the *Dlk1-Dio3* domain lncRNA, we inserted a transcription termination sequence (3×polyA) at the end of exon 1 of *Gtl2* using Easi-CRISPR technology (Figure 1A). As *Gtl2* is a maternally expressed gene, we maintained the population by crossing heterozygous male mice with wild-type (WT) DBA/2J females. Genotyping was performed using polymerase chain reaction (PCR) (Figure 1B). 

### 2.2. Dlk1-Dio3 Domain lncRNA Is Required for Normal Embryo Growth and Heart Development 

During sample collection, we found that the distribution of offspring genotypes did not conform to mendelian laws of inheritance [25]. Statistical results showed that maternal insertion mutant (MKI) and homozygous mutant (HOMO) embryos started to die at E13.5, with all of them dying at E16.5, and Parental insertion mutants (PKI) embryos grew and developed normally. MKI and HOMO mutant embryos exhibited edema when close to death (Figure 1C), suggesting a possible abnormality in embryonic heart development. Subsequently, we counted the heart rates of surviving embryos at E12.5, 14.5, and 16.5 within 20 s and found that the heart rates of MKI and HOMO embryos were significantly lower at the E14.5 stage (Figure 1D, Tables S1–S3, Video S1). By observing the ventral–dorsal images of the hearts of E12.5 and 14.5, we found obvious nodules on the ventricular surfaces of MKI and HOMO embryos at E12.5, and the phenomenon was more pronounced at E14.5 (Figure 1E, the white arrows point to the abnormalities). There are no significant differences between E16.5 PKI and WT embryonic hearts (Figure S2A). Photographs of the heart used for observational analysis are shown in Figure S1.

### 2.3. Dlk1-Dio3 Domain lncRNA Expression Deficiency Impairs Ventricular Wall Development

**Figure 2:**
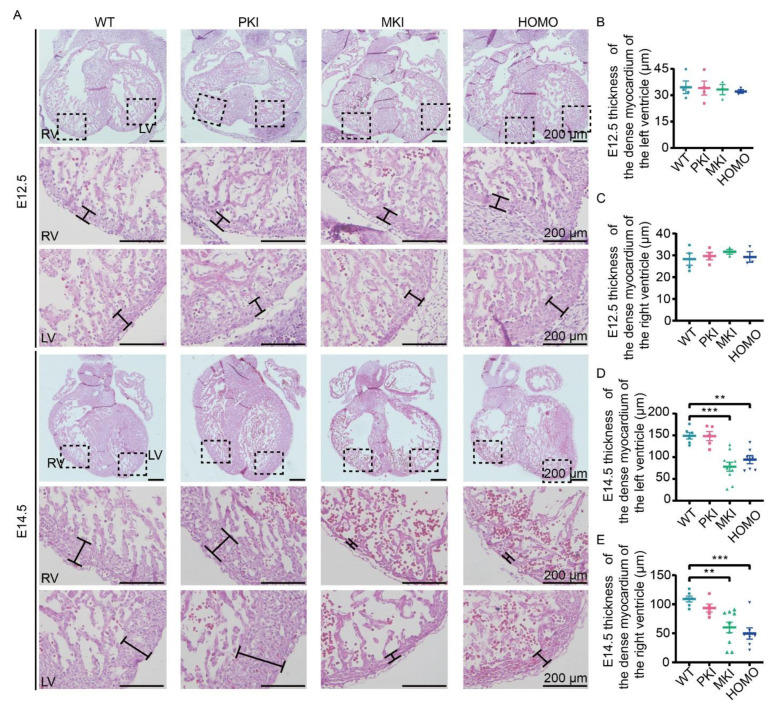
Histology of the E12.5 and E14.5 embryonic heart. (E12.5: WT n = 4, PKI n = 4, MKI n = 3, HOMO n = 3; E14.5: WT n = 6, PKI n = 5, MKI n = 10, HOMO n = 7) (**A**) H.E. staining of paraffin sections of E12.5 and E14.5 embryonic hearts. The dashed box shows the location of the enlarged image. Scale bars, 200 μm. (**B**,**C**) Statistical analysis of left and right ventricular wall thickness in E12.5 embryonic heart. (**D**,**E**) Statistical analysis of left and right ventricular wall thickness in E14.5 embryonic heart. Data are expressed as the mean ± SEM. ** *p* < 0.01; *** *p* < 0.001. RV, right ventricle; LV, left ventricle.

To further observe the histological changes in the embryonic heart after *Gtl2* polyA knock-in, we performed hematoxylin and eosin (H.E.) staining of heart sections from E12.5, 14.5, and 16.5 embryos. H.E. staining results showed no significant changes in PKI, MKI, and HOMO embryonic hearts at E12.5 (Figure 2A–C) and a significant decrease in left and right ventricular wall thicknesses of MKI and HOMO embryonic hearts at E14.5 (Figure 2A,D,E), suggesting incomplete ventricular myocardial densification. At E16.5, PKI embryonic hearts showed no significant changes (Figure S2B–D).

### 2.4. Expression of Genes within the Dlk1-Dio3 Domain in the Embryonic Heart

**Figure 3:**
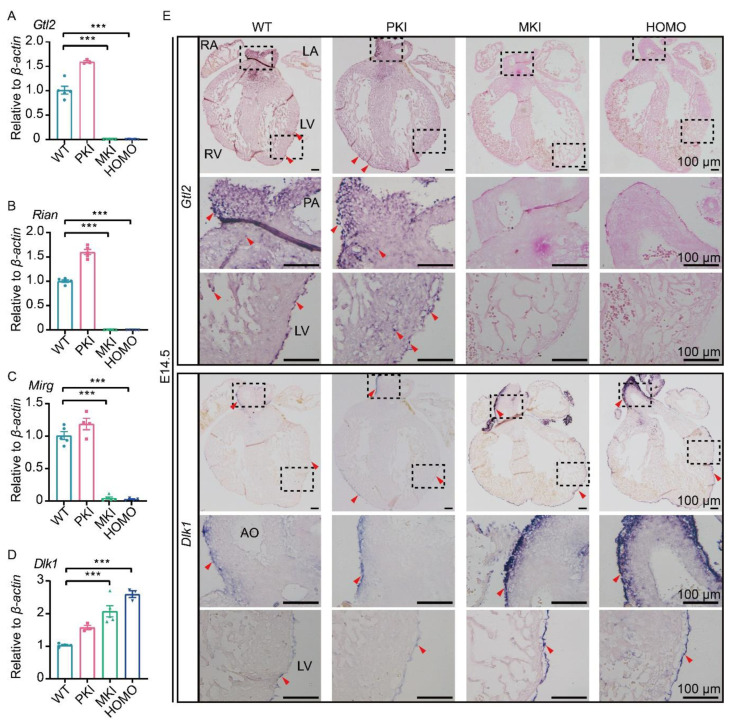
Expression of imprinted genes within the *Dlk1-Dio3* domain in E14.5 embryonic hearts. (**A**–**D**) Graphical representations of the expression levels of the four imprinted genes *Gtl2*, *Rian Mirg* and *Dlk1* at E14.5 (*Gtl2* WT n = 5, PKI n = 3, MKI, HOMO n = 5; *Rian* WT, PKI, MKI, HOMO n = 5; *Mirg* WT, PKI, MKI, n = 5, HOMO n = 3; *Dlk1* WT n = 4, PKI n = 3, MKI, n = 5, HOMO n = 3). The values represent the expression level relative to that of the *β-actin* expression level. Data are expressed as the mean ± SEM. *** *p* < 0.001. (**E**) ISH validated *Gtl2* and *Dlk1* expression changes of E14.5 embryonic heart. Scale bars, 100 μm. Red arrow, purple blue color indicating *Gtl2*-positive cells and *Dlk1*-positive cells. The dashed box shows the location of the enlarged image. RA, right atrium; LA, left atrium; RV, right ventricle; LV, left ventricle; AO, aorta; PA, pulmonary artery.

To explore the effect of *Gtl2* polyA knock-in on gene expression changes within the *Dlk1-Dio3* domain, we analyzed the expression patterns of E14.5 *Gtl2*, *Rian*, *Mirg* and *Dlk1* using quantitative reverse-transcription PCR (qRT-PCR) and in situ hybridization (ISH). The results of qRT-PCR showed that *Gtl2*, *Rian*, and *Mirg* were barely expressed in MKI and HOMO embryos, whereas *Dlk1* expression was upregulated in MKI and HOMO embryos by around 2-fold in the hearts of WT embryo (Figure 3A–D). The results of ISH showed that *Gtl2* and *Rian* were widely expressed in embryonic hearts. Both of them are expressed in the epicardium, ventricular wall, trabecular myocardium, muscular septum of the OFT, and the base and walls of the aorta and pulmonary artery of the embryonic heart. As in previous reports [26], *Dlk1* is mainly expressed in Epis. *Gtl2* and *Rian* were barely expressed in MKI and HOMO embryonic hearts, whereas *Dlk1* expression was upregulated (Figure 3E, Figure S3A). ISH results were consistent with the results of qRT-PCR in MKI and HOMO embryonic hearts.

### 2.5. ScRNA-Seq Analysis and Validation of the E14.5 Heart

**Figure 4:**
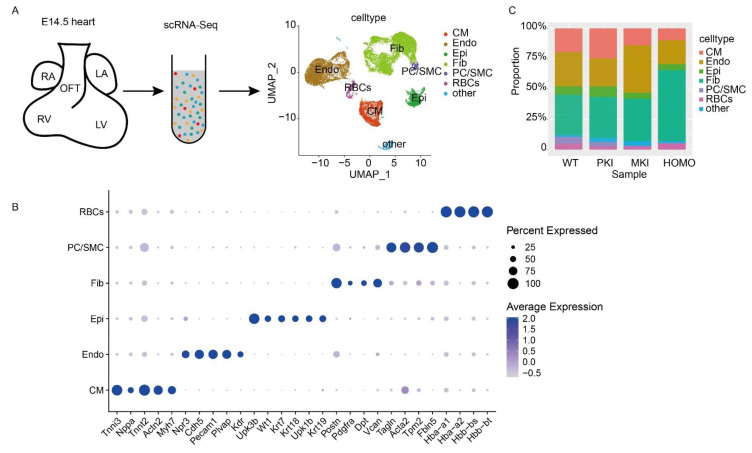
ScRNA-Seq analysis of E14.5 embryonic hearts. (**A**) Schematic of scRNA-Seq. (**B**) Expression dot plot of clusters and their respective markers. Dot size represents fraction of cells expressing the marker, and fill color corresponds to expression levels. (**C**) The relative contribution of each cell cluster within E14.5 embryonic heart. CM, cardiomyocyte; Endo, endothelial cells; Epi, epicardial cell; Fib, fibroblast cell; PC/SMC, pericyte/smooth muscle cells; RBCs, red blood cells.

ScRNA-seq is a powerful method to study cardiac development at the single-cell level [27,28]. In this study, we extracted a total of 55,660 cells from the four genotypes and, after quality control, screened 3975 cells from WT embryonic hearts; 3296 cells from PKI embryonic hearts; 9742 cells from MKI embryonic hearts; and 6207 cells from HOMO embryonic hearts for subsequent analysis. They were categorized into six major cell clusters by each cell-type marker (Figure 4A). CMs were labeled with *Tnni3* [29,30], *Nppa* [31], *Tnnt2* [29], *Actn2* [29], and *Myh7* [32] (Figure 4B). PCs/SMCs were labeled with *Tagln* [30,33], *Acta2* [30,33], *Tpm2* [34], and *Fbln5* [35] (Figure 4B). Epis were labeled with *Upk3b* [29,30], *Wt1* [29], *Krt7* [32], *Krt18* [32], *Upk1b* [28] and *Krt19* [30] (Figure 4B and Figure S4). Endos were labeled with *Npr3* [33], *Cdh5* [33], *Pecam1* [28], *Plvap* [29], and *Kdr* [29] (Figure 4B). Fibs were labeled with *Postn* [36], *Pdgfra* [28], *Dpt* [30], and *Vcan* [30] (Figure 4B). By analyzing the proportions of the major cell types in the four genotypes, the results showed a tendency for the proportions of CM, Epi, and PC/SMC cell clusters to decrease (Figure 4C). As seen by the expression of genes within the *Dlk1-Dio3* domain in each cell type, *Gtl2* was mainly expressed in the PC/SMC cell cluster, *Dlk1* was mainly expressed in the Epi cell cluster, and *Mirg* expression was low in all types of cell clusters in the heart (Figure S5). Enrichment analysis of all cellular differential genes of the four genotypes was performed. We focused on the differential gene clusters of MKI and HOMO with WT embryonic hearts (C1 and C5), the clusters where the protein-coding gene *Dlk1* and lncRNA *Gtl2* are located. Specifically, Gene Ontology (GO) enrichment such as the ECM-receptor interaction and translation at synapses were enriched by the upregulated genes. In contrast, the downregulated genes were enriched in heart valve morphogenesis and the extracellular matrix (Figure S6). 

**Figure 5:**
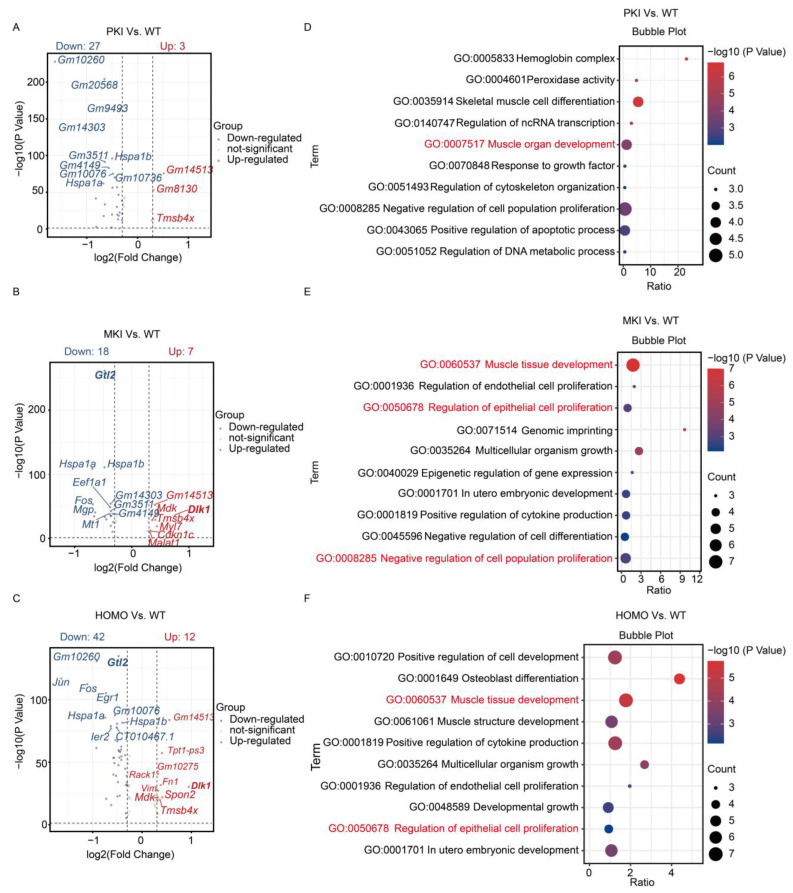
Bioinformatics analysis of epicardial cell clusters. (**A**–**C**) Volcano plot showing differentially expressed genes in the epithelial cell clusters between PKI, MKI, HOMO mutants and WT embryos. (**D**–**F**) GO enrichment analysis of differentially expressed genes in PKI, MKI and HOMO mutants of E14.5 with WT embryonic cardiac Epis. Important GO biological processes are shown in red.

Several articles have reported that Epis can differentiate into SMCs, Fibs, and other major cell types that make up the heart [3,37,38]; our focused analysis of Epis revealed that both MKI and HOMO embryonic hearts shared similar differently expressed genes, such as *Dlk1*, *Gm14513*, *Tmsb4x*, and *Gtl2*, compared to WT embryos (Figure 5A–C). Using GO analysis of the differential genes of MKI and HOMO embryonic Epis, we found that they could be enriched in biological processes such as muscle tissue development and regulation of epithelial cell proliferation (Figure 5D–F). 

**Figure 6:**
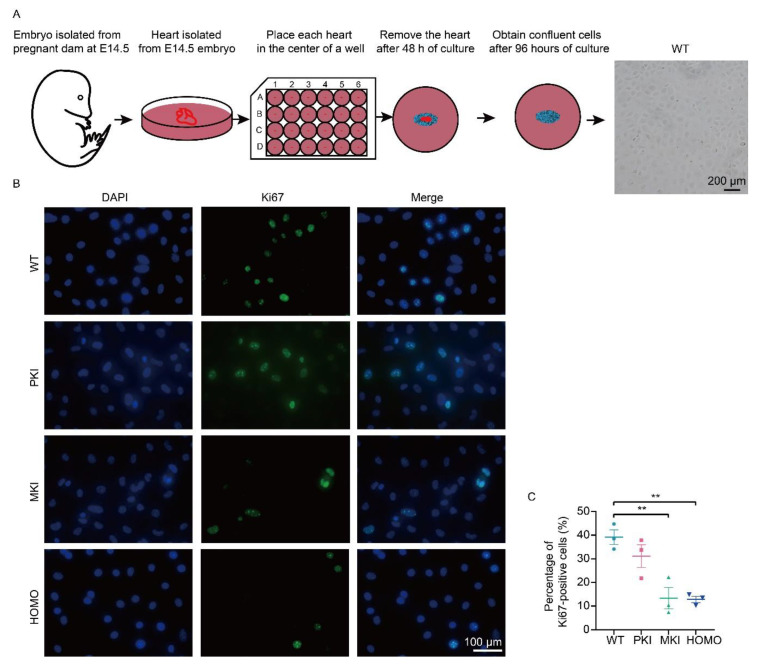
Isolation of primary Epis of various genotypes and evaluation of proliferative potential. (**A**) Schematic diagram of the workflow for primary epicardial cell isolation. Scale bar, 200 µm. (**B**) IF of primary Epis Ki67 (green) and DAPI (blue). Scale bar, 100 µm. (**C**) Percentage of Ki67-positive cells (n = 3). Data are expressed as the mean ± SEM. ** *p* < 0.01.

Based on the results of the above analysis, we extracted Epis from four genotype E14.5 embryos, and the operation procedure is schematically shown in Figure 6A. Cell purity was identified by using immunofluorescence (IF) and qRT-PCR, and the results showed high purity (Figure S7). This was then used for further study. IF results showed a decrease in the number of Ki67-positive cells in the Epis of MKI and HOMO mice (Figure 6B,C). Through immunohistochemical (IHC) observation of heart sections, we found the same expression trend of Ki67 (Figure S8). To clarify the status of epicardial development, we performed WT1 staining of the heart by immunohistochemistry, which showed that there was not an obvious defect in epicardial layer establishment in PKI, MKI, and HOMO embryos, but MKI and HOMO embryonic hearts had reduced expression of WT1 (Figure S9). Genes enriched for GO biological processes were quantitatively validated using extracted Epi samples. The results showed that *Dlk1* expression was upregulated in the Epis of MKI and HOMO embryos, which was approximately 2-fold that of the epicardium of WT embryos (Figure S10A). *Jun*, *Myl7* and *Fos* expression tended to be downregulated; however, the difference was not significant (Figure S10B–D). The expression of Dlk1 levels was verified by IHC, which showed increased expression in MKI and HOMO embryos (Figure S10E). The results showed that abnormal gene expression of the *Dlk1-Dio3* domain caused reduced proliferation of Epis. This may further affect the degree of cardiomyocyte densification, ultimately resulting in abnormal embryonic development or even death. 

## 3. Discussion

Our study shows that loss of *Dlk1-Dio3* domain lncRNA expression leads to defects in ventricular myocardial development in MKI and HOMO embryos, which began to die at E13.5, and all died at E16.5. In 2009, Tomohiro Kono’s team constructed a mouse model knocking out a genomic DNA deletion of about 9.8 kb genomic DNA length in exon *Gtl2* 1-5 [22]. In 2010, Anne Klibanski’s team constructed a mouse model with a knockout of a genomic DNA deletion of approximately 5.6 kb genomic DNA length in exon *Gtl2* 1-5 [23]. In 2019, Yunli Zhou’s team published a report by constructing two *Gtl2* deletion mouse models, *Meg3* (*Gtl2*) exon 1-4 deletion and *Meg3* (*Gtl2*) exon 2-4 deletion [24]. The different lengths of genomic DNA knockout fragments and the different genetic backgrounds of the above three models, coupled with the complexity of the regulatory mode of the *Dlk1-Dio3* domain and the diversity of maternal non-coding RNA action pathways, have also posed a major obstacle to exploring the function of this domain. The reason for the discrepancy between our results and previous studies may be due to the different model construction method used in this study. Gene silencing was obtained by inserting transcription termination fragments rather than by large knockouts, which may have reduced the discrepancy caused by large knockout differences [39,40]. This study provides a novel perspective for understanding the *Dlk1-Dio3* domain.

Although not yet confirmed, all maternally expressed genes within the *Dlk1-Dio3* domain have been shown to be initiated by the *Gtl2* transcription start site, where individual genes are acquired by post-transcriptional processing [41]. Our results show downregulation of lncRNA *Gtl2*, *Rian*, and *Mirg* expression in both MKI and HOMO embryonic hearts. *Gtl2* has been shown to play a role in senescence-mediated Endo dysfunction. Inhibition of *Gtl2* promotes sprouting of aged human vascular Endos (HUVECs) and improves blood flow in the ischemic hindlimb of aged mice [42]. LncRNA *Gtl2* directly or competitively binds to miRNAs and is involved in cell proliferation, apoptosis, and the epithelial–mesenchymal transition (EMT) process [43]. LncRNA *Rian* has been reported to reduce sepsis in CMs and attenuate myocardial ischemia–reperfusion damage by regulating the miR-17-5p/CCND1 axis [44]. No studies of the lncRNA *Mirg* associated with cardiac development or disease have been reported. The results of expression of major genes within the *Dlk1-Dio3* domain in various cell types of the heart show that the lncRNA *Mirg* is expressed at low levels in the heart. Our results also showed that miRNA-127, -409, -154, -495, and -300 expression is downregulated in MKI and HOMO embryonic hearts (Figure S3B–F). Although there is evidence and predictive analytics showing that miRNAs within the *Dlk1-Dio3* domain have a regulatory role in neonatal rat cardiomyocyte proliferation [45,46,47], they were expressed in low abundance in the embryonic heart during this period; they are mostly expressed in the brain and nervous system [48,49]. In addition, numerous miRNAs exist within the *Dlk1-Dio3* domain, and it is difficult to determine which of them are important for embryonic heart development. In summary, although the expression of the lncRNAs *Gtl2*, *Rian*, *Mirg*, and miRNAs was reduced in both MKI and HOMO embryonic hearts in this study, we focused on the analysis of the lncRNA *Gtl2*. Subsequently, we can construct novel animal models or extract primary cells to build cell lines, so that we can determine their functions in cardiac development one by one. In addition, the protein-encoding gene *Dlk1* within the *Dlk1-Dio3* interval showed a trend toward elevated expression in the hearts of MKI and HOMO embryos, with expression approximately 2-fold that of the hearts of WT embryos. One study showed that *Gtl2* expression prevents chromatin activation on the maternal *Dlk1* promoter [50]. In a recently published report, it is lncRNA *Gtl2*, but not *Rian*, small RNAs, that suppresses *Dlk1* expression on the maternal chromosome during mouse embryonic stem cell differentiation [51]. This could explain the result that *Dlk1* expression is upregulated after *Gtl2* insertion of a transcription termination signal. *Dlk1* is highly expressed during embryogenesis. As an atypical Notch ligand, numerous studies have shown that *Dlk1* can affect cell proliferation, differentiation, and EMT transformation through the Notch pathway and has a function in Endos to stimulate blood vessel development [52,53]. Although not the primary focus of this research, one study reported that *Dlk1* regulates *Notch1* activation during mammalian heart development in conjunction with other target genes of *ZFP57*, thereby affecting the development of the ventricular wall and cardiac chambers [26]. In mice, although the overall phenotype of the heart is modest, a marked difference was observed in the overall structure of the large coronary vessels of the *Dlk1*(−/−) mutant hearts, with a reduction in the number of non-myocytes in the ventricles of the mice [54]. Collectively, we hypothesized that ventricular wall hypoplasia caused by insertion of a transcriptional termination signal at *Gtl2* might be due to an upregulation in *Dlk1* expression. It is also important to note that in our model, since the embryo is in the process of progressive death at E14.5, this could also be one of the reasons for the phenotype of abnormal heart development that we observed.

In recent years, the rapid development of scRNA-seq technology has enabled the use of single-cell transcriptional profiling to explore cellular heterogeneity within the heart and improve our understanding of cardiac differentiation processes [55,56]. Classification of cells by known markers enables a more objective analysis of the contribution of each type of cell during cardiac development [28,57]. We analyzed the enrichment of all cellular differential genes for the four genotypes. The results showed that MKI and HOMO embryonic cardiac abnormalities were associated with ECM receptor interactions and extracellular matrix processes. The ECM forms a highly dynamic and plastic milieu that has an active and crucial role in the regulation of Epis. Perturbation of the expression of ECM components, or alterations in the signaling pathways that regulate their production, can lead to cardiac malformations [58]. At E9.5 in the mouse, cells derived from the proepicardium attach to the outer surface of the myocardium and subsequently spread over the heart to form the epicardial epithelium. Epis then undergo an epicardial EMT. This epicardial EMT generates a population of epicardially derived mesenchyme. A subset of epicardium-derived cells (EPDCs) subsequently migrate into the ventricular myocardial walls where they remain and reside as interstitial fibroblasts, differentiate into coronary smooth muscle cells, or possibly become coronary endothelium or cardiomyocytes [59,60]. Single-cell analysis showed a trend toward a decrease in the proportion of CM, PC/SMC, and Epi clusters in MKI and HOMO mutant embryos, which is consistent with our previously observed phenotype. An increasing number of studies suggests that the epicardium is a critical tissue for cardiac development as epicardium-derived signals must coordinate the development of the coronary vasculature with myocardial growth [13,38]. Therefore, we extracted E14.5 embryonic heart Epis for further study. Ki67 is a marker commonly used to assess cell proliferation [61]. After Ki67 IF of primary cells, the results showed reduced proliferation of Epis in MKI and HOMO mutant embryos. In addition, we performed IHC of embryonic hearts with WT1, and the results showed no abnormalities in epicardial formation in PKI, MKI, and HOMO embryonic hearts, but there were fewer WT1-positive cells in the ventricular myocardium of the MKI and HOMO embryonic heart. Previously, an article reported that these WT1-positive cells present in the subepicardium and ventricular myocardium may be Endos or coronary endothelial cells of epicardial origin [62]. The attenuation of WT1-positive signals in the hearts of MKI and HOMO embryos suggests that abnormalities may have occurred in the process of migration and differentiation of the epicardium to the interior of the heart. This may be the cause of ventricular wall hypoplasia in MKI and HOMO embryos. Epis have important contributions to all types of cell lines during cardiac development, and the reduced proliferation of Epis has certain effects on the development of other cells, such as PC/SMC, or Endo, CM, and in the future, the exact cause of the cardiac developmental abnormality in this model can be investigated and reported through tracking the Epi differentiation process by constructing conditional knockout mouse models. Biological processes showing differential gene enrichment in MKI and HOMO mutant embryos by differential GO enrichment analysis of clusters of Epis are “Muscle tissue development” and “Regulation of epithelial cell proliferation”. For both biological processes, we quantified the genes with extracted Epis samples, which showed a significant upregulation in *Dlk1* expression and an insignificant decrease in *Jun*, *Myl7*, and *Fos* gene expression. We surmise that this results from the reduced of Epi proliferation caused by elevated *Dlk1* expression in the epicardium. Epis, one of the progenitor cells of cardiac development, have received increasing and widespread attention in recent years. Increasing evidence suggests that pathways regulating epicardial development are reactivated during cardiac regeneration [63]. Therefore, understanding the factors affecting the proliferation of Epis can not only provide more information for exploiting their potential during cardiac development but also for targeting Epis to regulate the heart against disease injury. 

In fact, because the model mice are global *Gtl2* polyA knock-in, ventricular wall dysplasia is not the only cause of embryonic death in MKI and HOMO embryos. We have reported that, in the present model, the junctional zones of the placenta are expanded, and labyrinth areas are reduced [25]. The death of MKI and HOMO embryos was due to developmental disorders of multiple organs. 

Overall, our results suggest that *Dlk1-Dio3* domain lncRNA expression is downregulated and *Dlk1* expression is upregulated in the *Gtl2* polyA knock-in model. This further affects the proliferation of Epis, ultimately causing ventricular wall dysplasia in the embryonic heart. Our findings reveal the importance of lncRNAs within the *Dlk1-Dio3* domain during cardiac development and provide novel information for further studies of prenatal pathology in mice and humans.

## 4. Materials and Methods

### 4.1. Mouse Breeding, Timed Pregnancy Mating, and Genotyping

The animal testing protocol was approved by the Harbin Institute of Technology Animal Care and Use Institutional Committee (IACUC). The review number is IACUC-2023066. The F0 mice have a genetic background of C57/B6N and DBA/2J. In this study, Easi-CRISPR was used to insert a 3×polyA sequence (147 bp) at the end of exon 1 of the *Gtl2* gene to block the transcription of the maternal RNA in the *Dlk1-Dio3* imprinted region. The chromosomal position of insertion: mouse (GRCm38/mm10) chr12: after 109,541,067. The sequences of single-guide RNA (sgRNA) and single-strand DNA donor (ssDNA donor) were described in another of our studies [25]. Mutant mouse lines were established by crossing F0 males with DBA/2J females. Heterozygous mutants were maintained only when the mutation was inherited from the father because the MKI mutant died during embryonic life. PKI and MKI mutants were generated by crossing WT and PKI mutants with each other. HOMO mutants were obtained by crossing male PKI mutants with female PKI mutants. The half-day pregnancy (E0.5) was defined as midday of the day when the vaginal plug was found. The targeted allele was identified using PCR using gDNA with primers *Gtl2*-543 bp F (5′-CAGCCCCTAGCACAGAAGAC-3′) and *Gtl2*-543 bp R (5′-ATTTTATGGGGCTTGCGGGA-3′). For heart rate data collection, to ensure sample freshness and to minimize the effect on embryonic heart rate caused by prolonged time away from the uterus, we kept the embryos warm in phosphate-buffered saline (PBS) solution in a 37 °C incubator and immediately counted the heart rates of the embryos for 20 s [64].

### 4.2. Histological Analysis and Quantification of Ventricular Wall Thickness

Embryonic or cardiac samples isolated from pregnant mice were fixed in 4% paraformaldehyde (PFA) solution overnight and embedded in paraffin. Transverse serial sections of 5 μm thickness were stained with H.E. All slides were imaged using ZEN (version 3.9, ZEISS, Jena, Germany) software. Ventricular wall thickness was measured for each genotype using ImageJ software version 1.46r (NIH, Bethesda, MD, USA). For each parameter, three measurements were taken along the lateral sides of each ventricle and averaged individually.

### 4.3. RNA ISH with Digoxigenin Labeling

Target gene amplification primers are shown in Table S4. Amplified target gene fragments were ligated into the pBlueScript II KS (+/−) vector. After digestion, the purified linear vector was used to synthesize the template for the RNA probe. The probes were prepared using the DIG RNA labeling kit (10881767001, Roche, Mannheim, Germany). Embryonic or cardiac samples were paraffin-embedded after fixation in 4% PFA solution for 24 h. The paraffin sections with 10 μm thickness were dewaxed and rehydrated. Sections were digested with proteinase K (C2449, Alpha biotech, Shanghai, China) for 15 min at room temperature. Proteinase K action terminated with glycine. Sections were incubated with acetylation solution for 15 min at room temperature. Sections were prehybridized in hybridization buffer at 65 °C and were incubated with RNA probes (200 ng/mL in hybridization buffer) at 65 °C overnight. Sections were washed with 0.2×SSC, blocked with blocking buffer at room temperature, and incubated with alkaline phosphatase-conjugated anti-DIG antibodies (1:5000 dilution, 11093274910, Roche, Mannheim, Germany) at 4 °C overnight. Hybridization signals were detected with NBT/BCIP stock solution (11681451001, Roche, Mannheim, Germany). All sections were stained with eosin and sealed for photographic observation. 

### 4.4. RNA Isolation and qRT-PCR

Primer sequences used for qRT-PCR are shown in Table S5 of the Supplementary Material. Total RNA was isolated from embryonic hearts using Trizol reagent (9109, Takara, Osaka, Japan). For mRNA and lncRNA, complementary DNA (cDNA) was synthesized using PrimeScript™ RT Master Mix (RR047A, Takara, Osaka, Japan) according to the manufacturer’s instructions. Reverse transcription for miRNA was performed using Mir-X™ miRNA First-Strand Synthesis (638314, TakaRa, Osaka, Japan). qRT-PCR was performed using the ViiA™ 7 Real-Time Fluorescence PCR System (Thermo Fisher Scientific, Waltham, MA, USA). Data were analyzed by relative gene expression using the 2^−ΔΔCT^ method, and gene expression data were normalized using the housekeeping gene *β-actin* and expressed relative to the WT genotype. *U6* was used as the internal reference gene for miRNA. 

### 4.5. scRNA-Seq Analysis

The hearts of WT, PKI, MKI, and HOMO embryos (one embryonic heart per genotype) at E14.5 were dissected and provided in Tissue Preservation Solution to Singleron Biotechnologies (Nanjing, China) for scRNA-seq. Single-cell suspensions were loaded into microfluidic devices, and scRNA-seq libraries were constructed using the GEXSCOP Single-Cell RNA Library Kit (Singleron Biotechnologies) and sequenced on an Illumina HiSeq X instrument (Illumina, San Diego, CA, USA). Raw sequencing data were handled using the CellRanger software version 1.1.0. All reads were aligned to the mouse GRCm38 genome, and genes were counted using single-cell expression count matrices and analyzed using the R package Seurat (4.3.0) [65]. Genes with at least one feature count in more than three cells were selected for subsequent analysis. Cells were filtered by gene counts between 200 and 3000. Cells with more than 5% of mitochondrial content were removed. Normalization was performed using the LogNormalize function, with the scale factor at 10,000. The ScaleData function with all genes was used to scale the data. We performed principal components analysis (PCA), and 10 significant principal components were used as the input for graph-based clustering. Clustering of heart cells was performed in Seurat at a resolution of 0.2. UMAP was used for two-dimensional visualization of the multi-dimensional dataset. The identities of the resultant clusters were inferred using literature-derived marker genes. 

Differentially expressed genes (DEGs) between the MKI and WT, PKI and WT, and HOMO and WT groups were calculated within each cell type using the FindAllMarkers function, using the Wilcoxon rank sum test to identify significant differences. Genes were deemed differentially expressed if they had an adjusted *p*-value less than 0.05 and an absolute fold change greater than 0.25. GO analysis was performed using Metascape https://metascape.org (accessed on 28 December 2023) and visualization performed with Hiplot https://hiplot.com.cn (accessed on 19 December 2023).

### 4.6. Isolation and Culture of Mice Epis

At E14.5, the embryo was dissected, and the uterus was placed in sterile PBS, which was pre-cooled on ice. The embryonic ventricles were removed under a body microscope using ophthalmic forceps. The ventricles were subsequently inoculated in 1% gelatin-coated 24-well petri dishes with Dulbecco’s Modified Eagle Medium (DMEM) containing penicillin, streptomycin, and 10% fetal bovine serum (FBS). The isolated ventricles were gently pressed onto the ventricular tissue with a sterile 1% gelatin-coated slide to adhere the tissue to the bottom of the dish, which was then incubated at 37 °C under 5% CO_2_. After 24 h, Epi monolayers grew out from the edge of the tissue block. After 48 h, the ventricular tissue was removed and incubation was continued to the appropriate time point for subsequent experiments. Fresh medium was added to the well plates every three days.

### 4.7. IF and IHC

After cell crawls were fixed with 4% PFA, treated with 0.2% Triton-100, and blocked with 4% bovine serum albumin (BSA)/PBS, primary antibodies WT1 (1:300 dilution, SDT-R076, Starter, China) and Ki67 (1:400 dilution, HGS-S239, Acrobiosystems, Beijing, China) were incubated at 4 °C overnight. After incubation a fluorescent secondary antibody (1:500 dilution, A11034, A11036, Invitrogen, Carlsbad, CA, USA) for 1 h, the cell crawls were incubated with DAPI (1:1000 dilution), washed three times with PBS, and sealed with anti-fluorescence quencher (BL701A, Biosharp, Beijing, China). Images were captured using an Agilent (Axio Zoom.V16, Santa Clara, CA, USA) microscope within 24 h of sealing, and cell counts were performed manually. For tissue immunohistochemistry, sections were 10 μm thick. After antigen repair, 5% H_2_O_2_ was incubated for 10 min. After blocking with 4% BSA/PBS, Ki67 (1:100 dilution, HGS-S239, Acrobiosystems, Beijing, China) and Dlk1 (1:500 dilution, ab210471, Abcam, Cambridge, UK) were incubated at 4 °C overnight. Horseradish peroxidase (HRP) secondary antibodies (1:5000 dilution, Beyotime Biotechnology, Shanghai, China) were incubated under room temperature for 1 h, and HRP detection was performed using a DAB peroxidase substrate detection kit (ZLI-9019, ZSGB-Bio, Beijing, China). All sections were imaged using ZEN (ZEISS) software.

### 4.8. Statistical Analysis

Statistical analyses were performed using GraphPad Prism 8.0 (Graphpad, Boston, MA, USA). All results are reported as the mean ± standard error of the mean (SEM) of at least three independent samples. One-way analysis of variance (ANOVA) was performed for statistical comparisons of data, followed by Tukey’s multiple comparison test. The results with * *p* < 0.05 were considered statistically significant in ANOVA.

## Data Availability

The article contains the data supporting its conclusions. The data utilized in this study can be obtained from the corresponding author upon request.

## Supplementary Materials

The following supporting information can be downloaded at: https://www.mdpi.com/article/10.3390/ijms25158184/s1.
