# Supplementary material for: LncRNAs in the Dlk1-Dio3 Domain Are Essential for Mid-Embryonic Heart Development
Source: Int J Mol Sci. 2024 Jul 26;25(15):8184. doi: 10.3390/ijms25158184 (PMC11311489; doi:10.3390/ijms25158184)
Supplement: Supplementary file 1 [file ijms-25-08184-s001.zip › Supplementary Material.pdf]

Supplementary Material

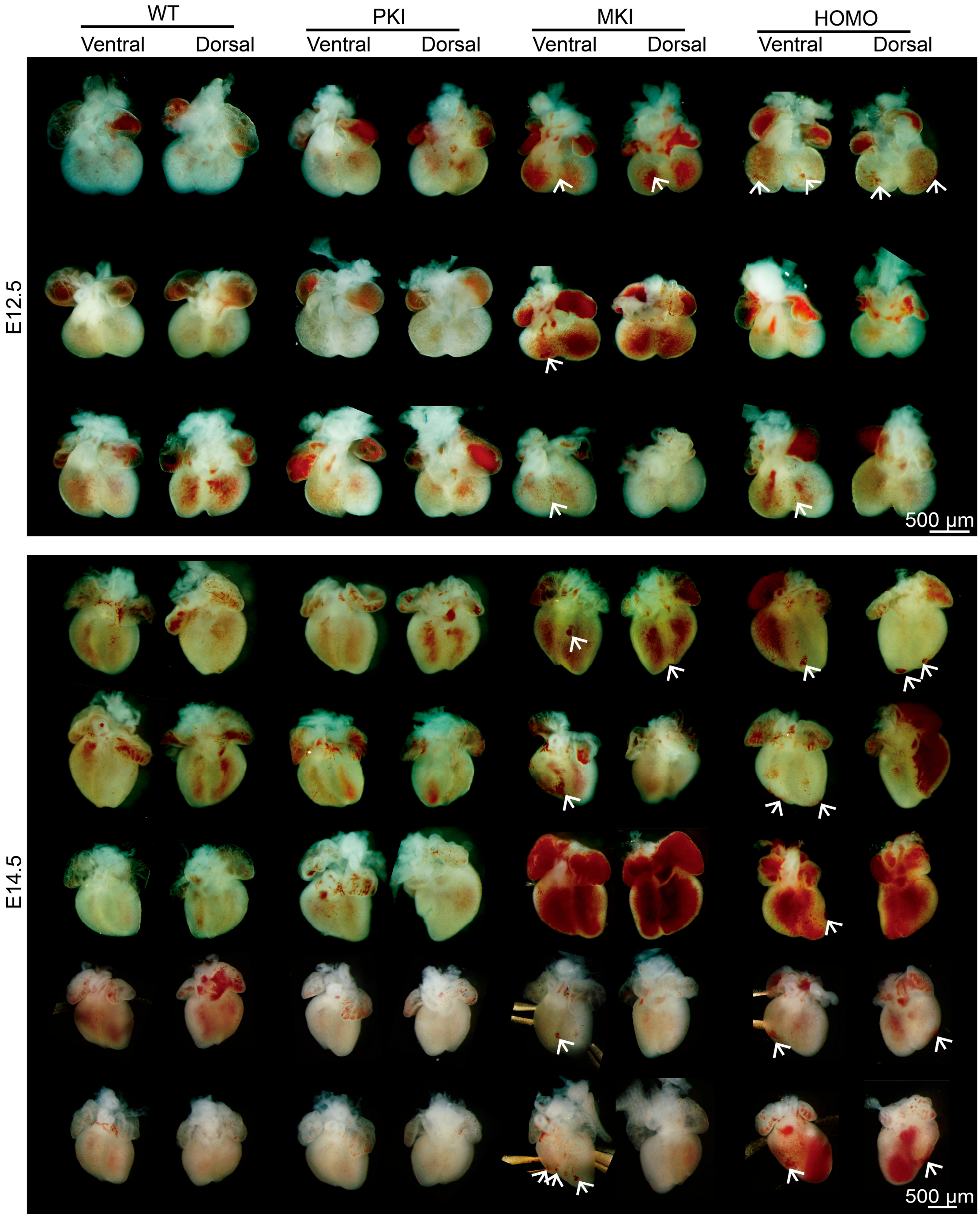

Figure S1 Photographs of E12.5 and 14.5 hearts used for analysis. Scale bar: 500 µm. (White arrows point to anomalies)

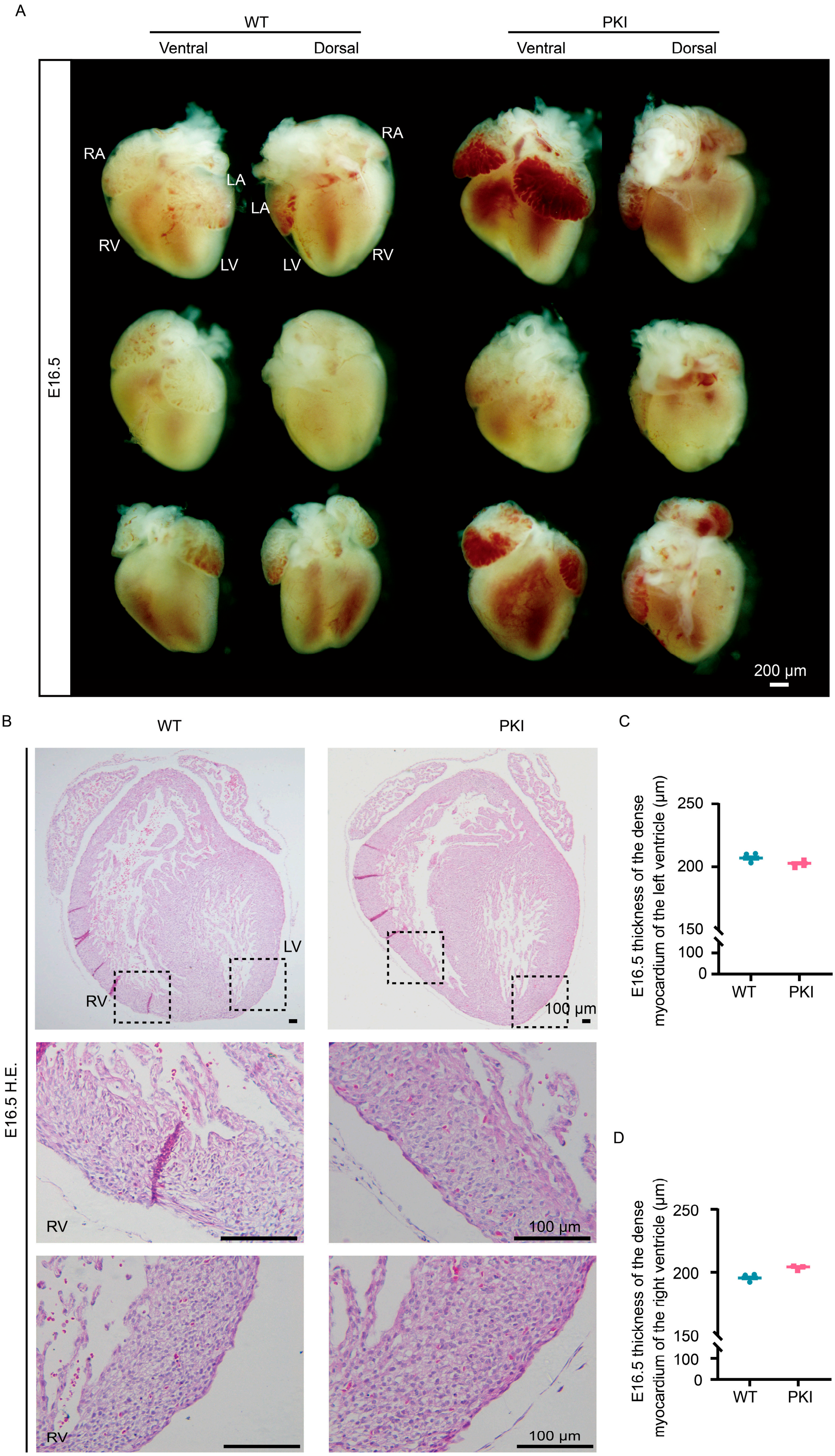
A
WT
PKI
Ventral
Dorsal
Ventral
Dorsal
E16.5
RA
LA
RV
LV
200 μm
B
WT
PKI
E16.5 H.E.
LV
RV
100 μm
C
E16.5 thickness of the dense myocardium of the left ventricle (μm)
250
200
150
100
0
WT
PKI
D
E16.5 thickness of the dense myocardium of the right ventricle (μm)

Figure S2 Photographs of E16.5 hearts used for analysis and H.E. histological analysis of E16.5 embryonic heart (n=3). (A) Photograph of E16.5 embryonic heart. Scale bar: 200 μm. (B) H.E. staining of paraffin section of E16.5 embryonic heart. The dashed box shows the location of the enlarged image. Scale bar: 100 μm. (C, D) Statistical analysis of E16.5 left and right ventricular wall thickness.

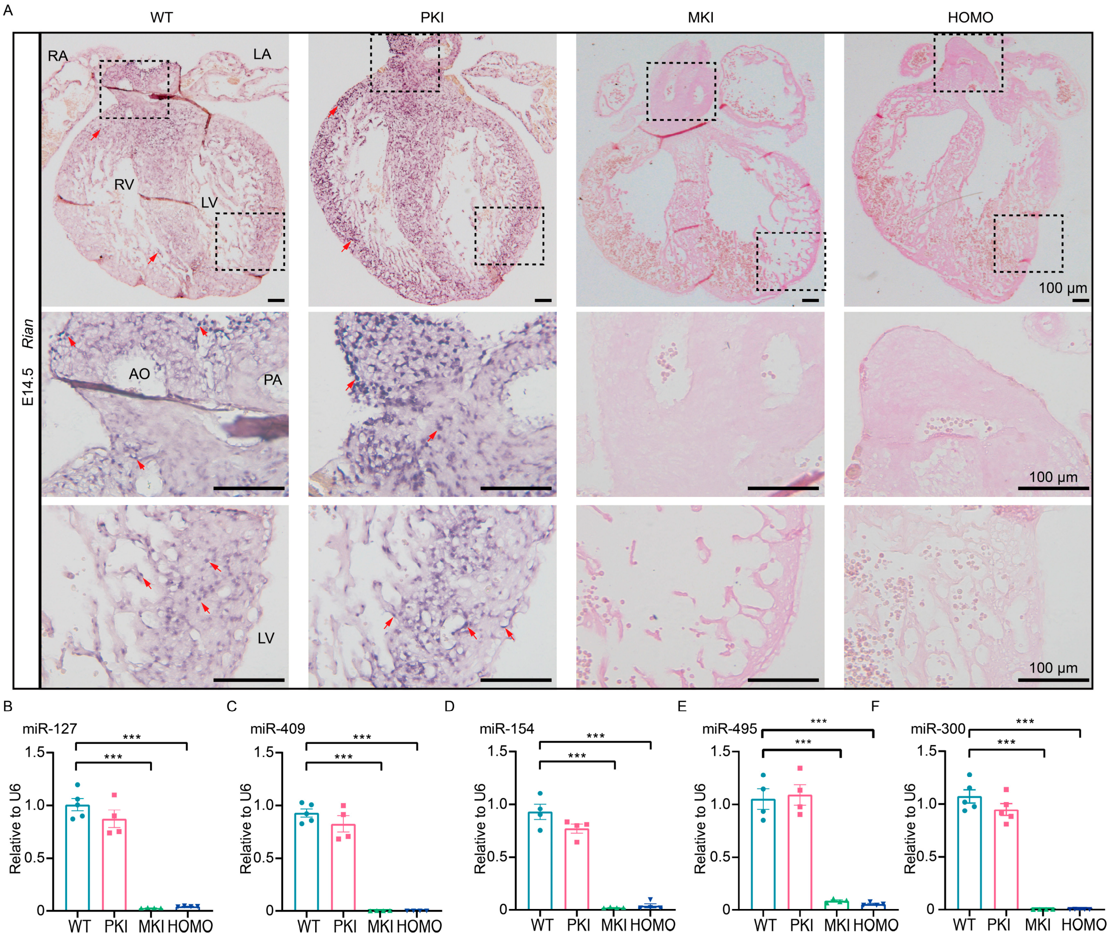

Figure S3 (A) *In situ* hybridization validated *Rian* expression changes of E14.5 embryonic heart. Scale bar: 100 μm. Red arrow, purple blue color indicating *Rian*-positive cells. The dashed box shows the location of the enlarged image. (B-F) Graphical representations of the expression levels of miR-127, 409, 154,495 and 300 at E14.5 (miR-127 WT n=5, PKI, MKI, HOMO n=4; miR-409 WT n=5, PKI, MKI, HOMO n=4; miR-154 n=4; miR-495 n=4; miR-300 WT, PKI n=5, MKI n=4, HOMO n=5). The values represent the expression level relative to that of the *U6* expression level. Data are expressed as the mean ± SEM. *** $p < 0.001$.

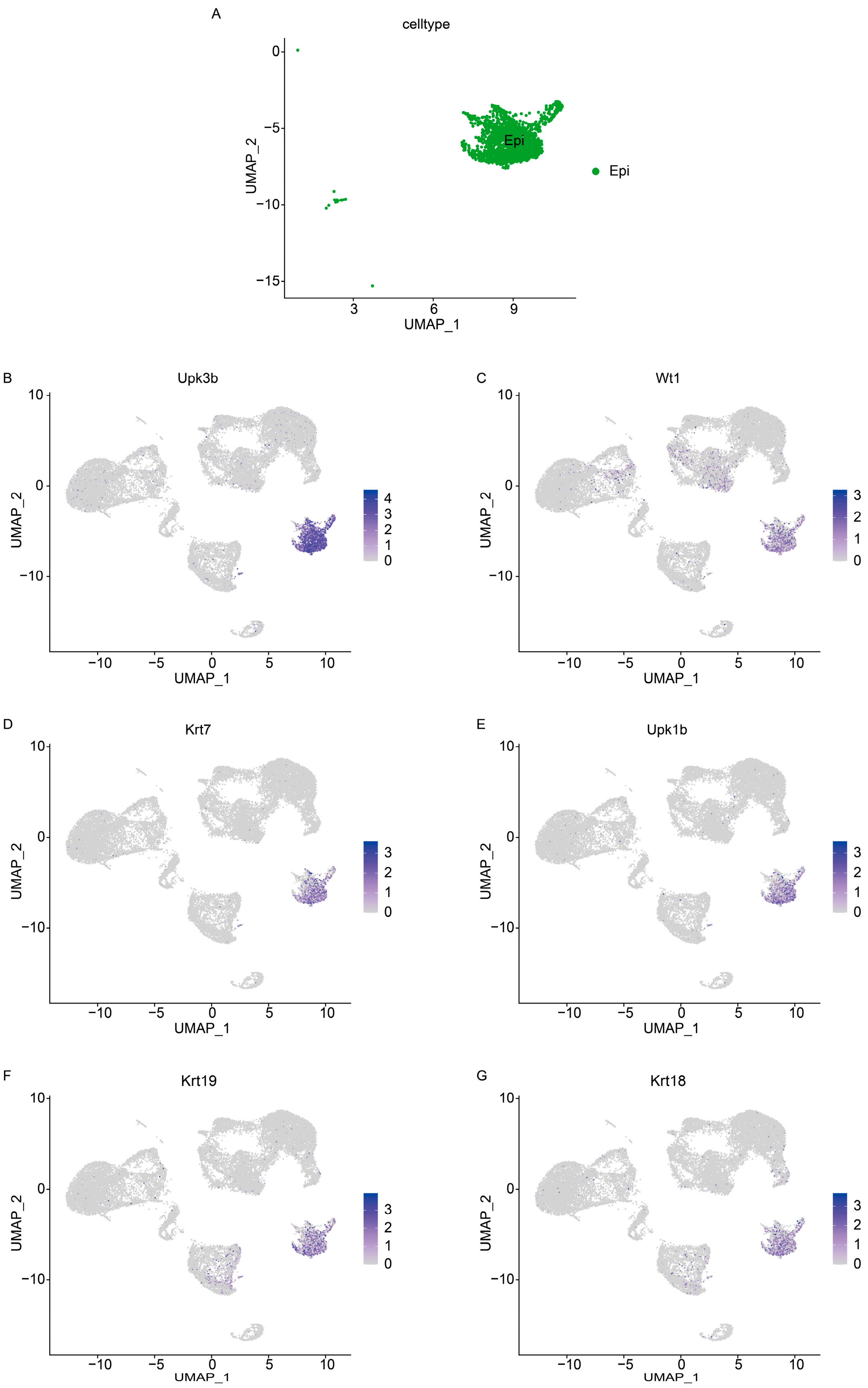
A
celltype
Epi
UMAP_1
UMAP_2
B
Upk3b
C
Wt1
D
Krt7
E
Upk1b
F
Krt19
G
Krt18

Figure S4 UMAP depicting *Upk3b*, *Wt1*, *Krt7*, *Upk1b*, *Krt19*, *Krt 18* transcript levels in cells mapped to the epithelial cell cluster.

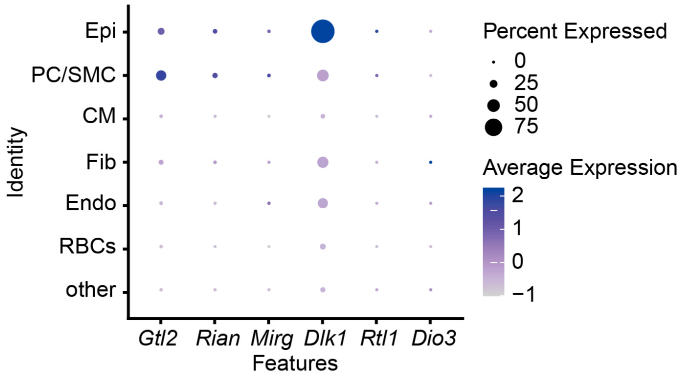

Figure S5 Expression of genes within the *Dlk1-Dio3* domain in various cell types.

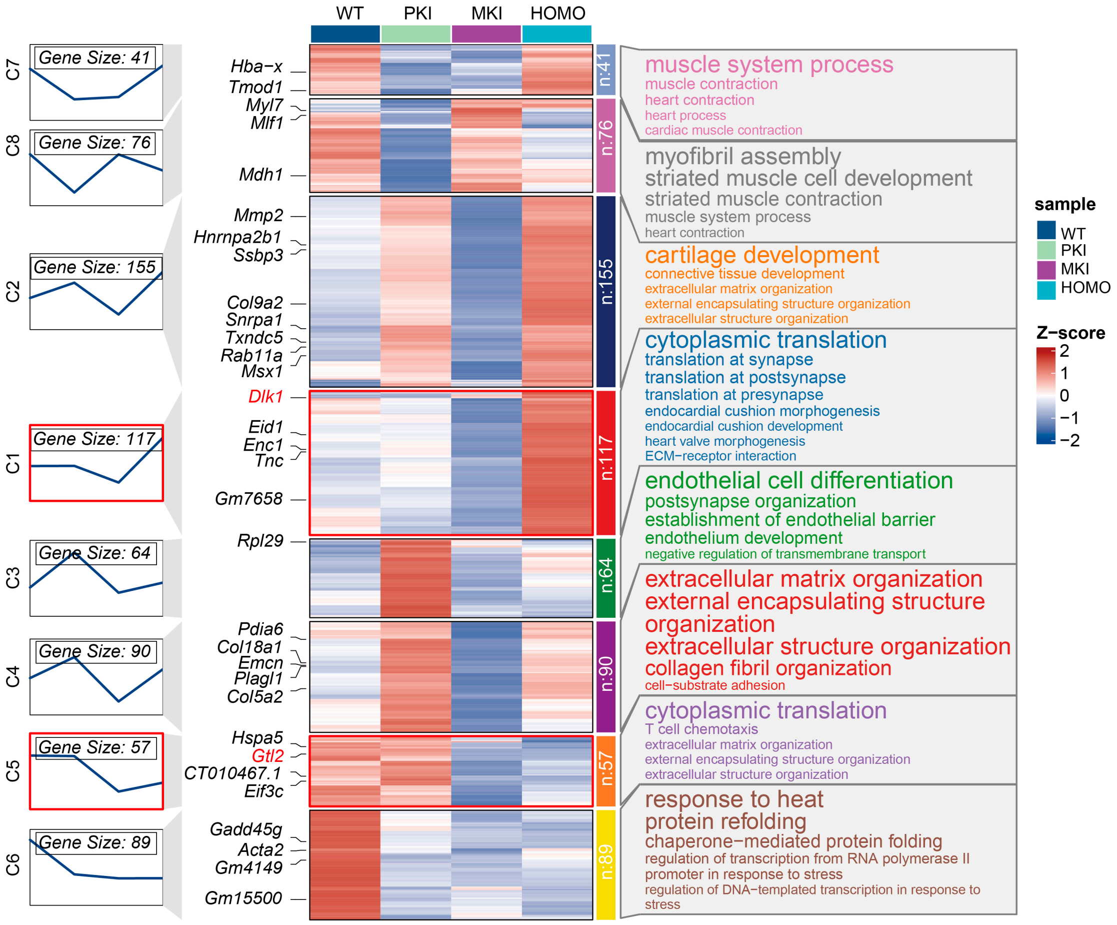

Figure S6 Enrichment analysis of differential genes

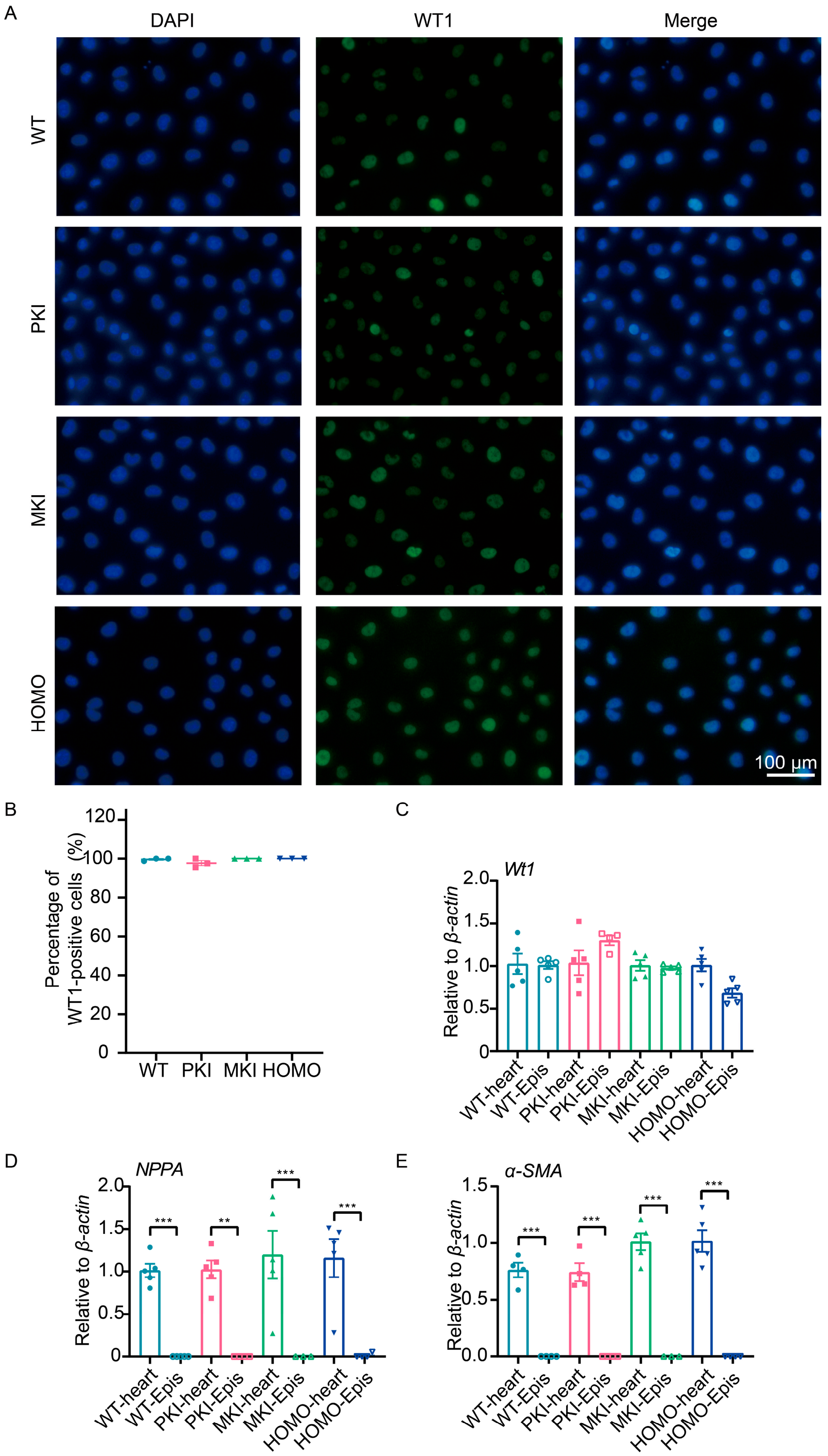

Figure S7 Identification of primary cell (A) Immunofluorescence of primary epicardial cells WT1 (green) and DAPI (blue). Scale bar, 100 μm. (B) Percentage of WT1-positive cells

(n=3). (C) qRT-PCR analysis of epicardial marker (*Wt1,* WT-heart n=5, WT-Epis n=5, PKI-heart n=5, PKI-Epis n=4, MKI-heart n=5, MKI-Epis n=5 HOMO-heart n=5, HOMO-Epis n=5), (D) qRT-PCR analysis of cardiomyocyte marker (*Nppa,* WT-heart n=5, WT-Epis n=5, PKI-heart n=5, PKI-Epis n=4, MKI-heart n=5, MKI-Epis n=3 HOMO-heart n=5, HOMO-Epis n=4), (E)qRT-PCR analysis of smooth muscle cell marker (*α-SMA,* WT-heart n=4, WT-Epis n=4, PKI-heart n=4, PKI-Epis n=4, MKI-heart n=5, MKI-Epis n=5 HOMO-heart n=5, HOMO-Epis n=4) expression levels. The values represent the expression level relative to that of the *β-actin* expression level. Data are expressed as the mean ± SEM. ** $p < 0.01$; *** $p < 0.001$

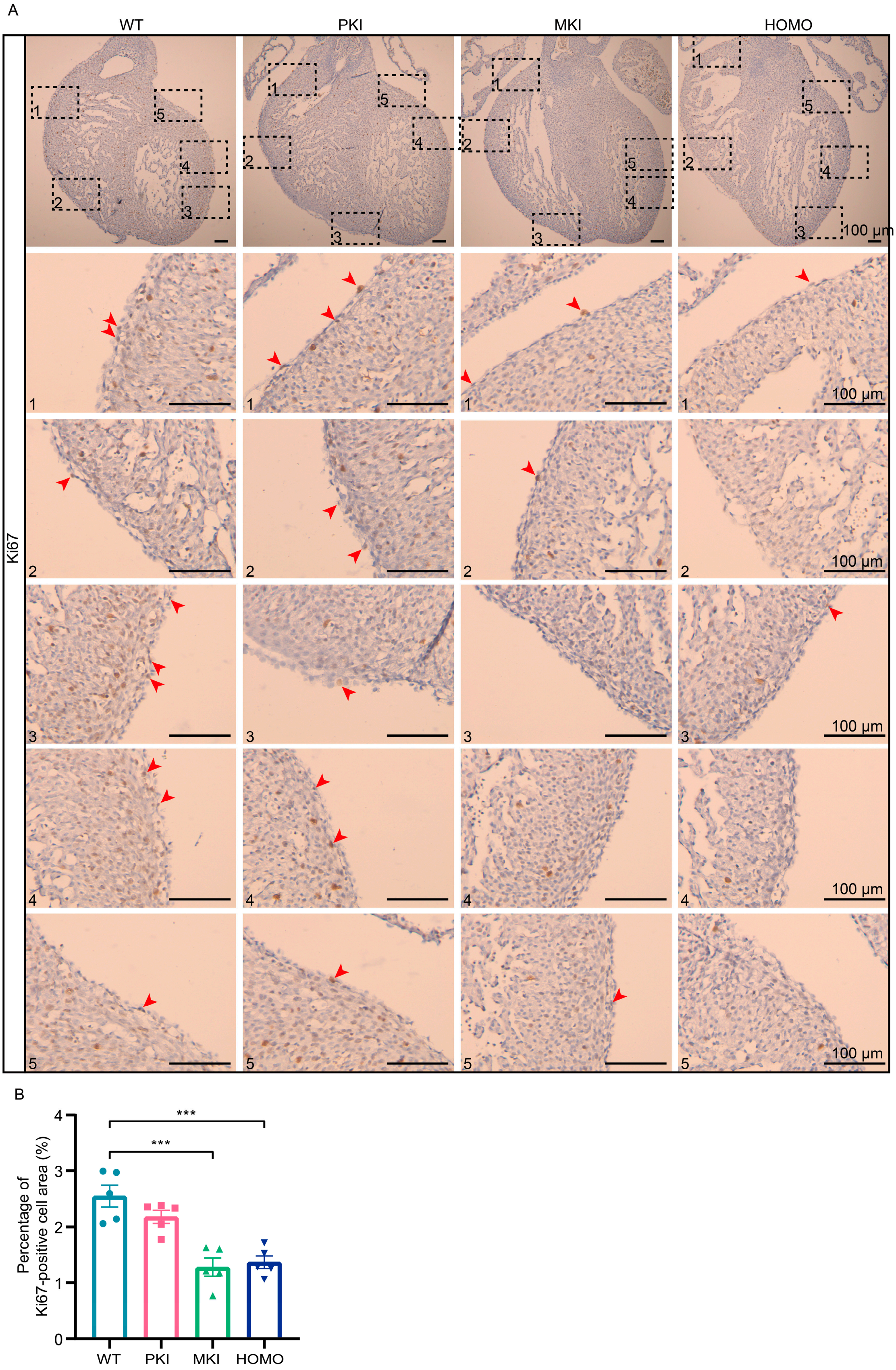

Figure S8 IHC results of Ki67 in E14.5 embryonic hearts. (A) IHC showing Ki67 expression. Scale bar: 100 μm. (B) Statistical on the percentage of tissue area occupied by Ki67-positive cells in five randomly selected areas. Data are expressed as the mean ±

SEM. *** $p < 0.001$.

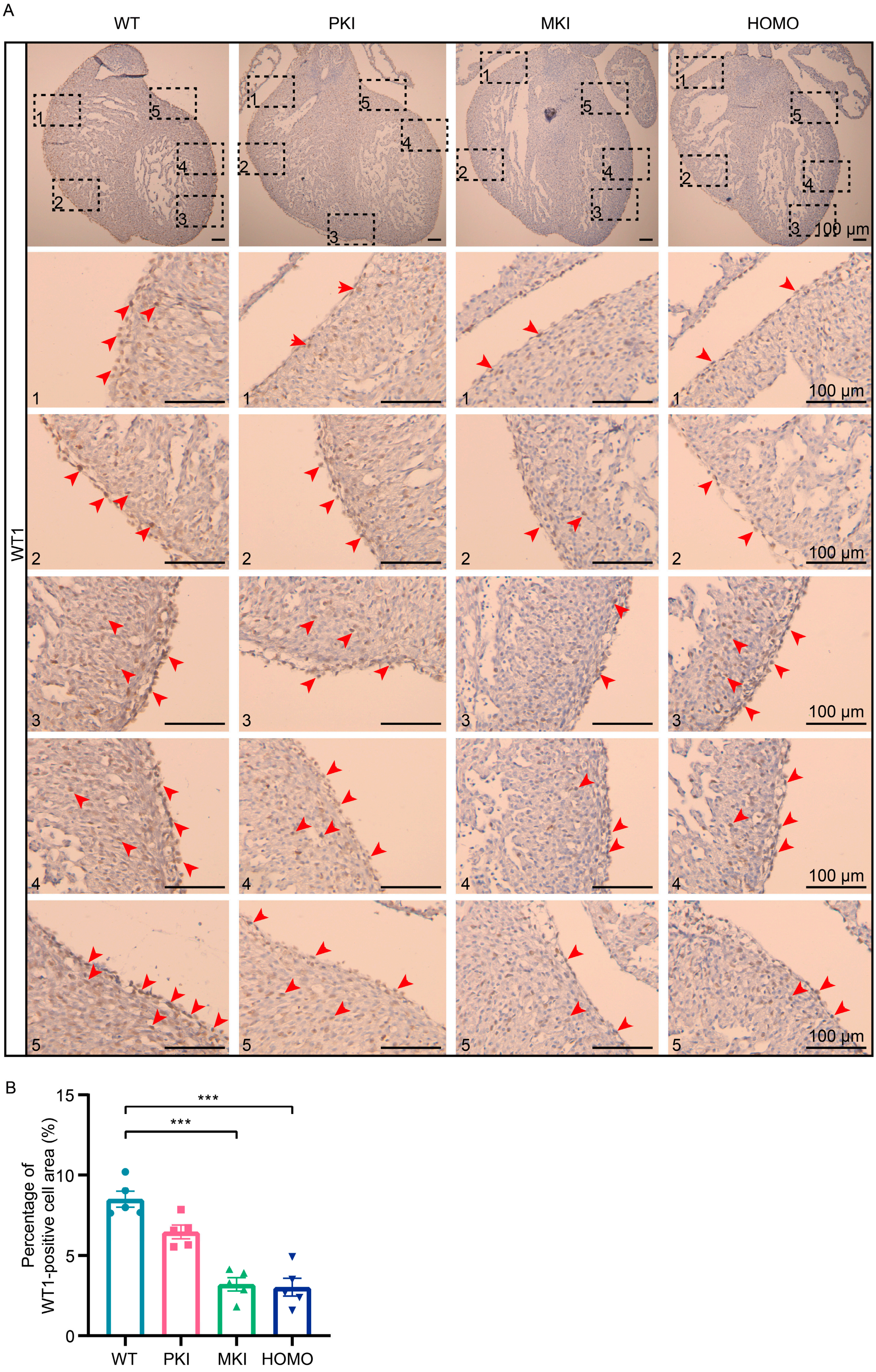

Figure S9 IHC results of WT1 in E14.5 embryonic hearts. (A) IHC showing WT1

expression. Scale bar: 100 μm. (B) Statistical on the percentage of tissue area occupied by WT1-positive cells in five randomly selected areas. Data are expressed as the mean ± SEM. *** $p < 0.001$.

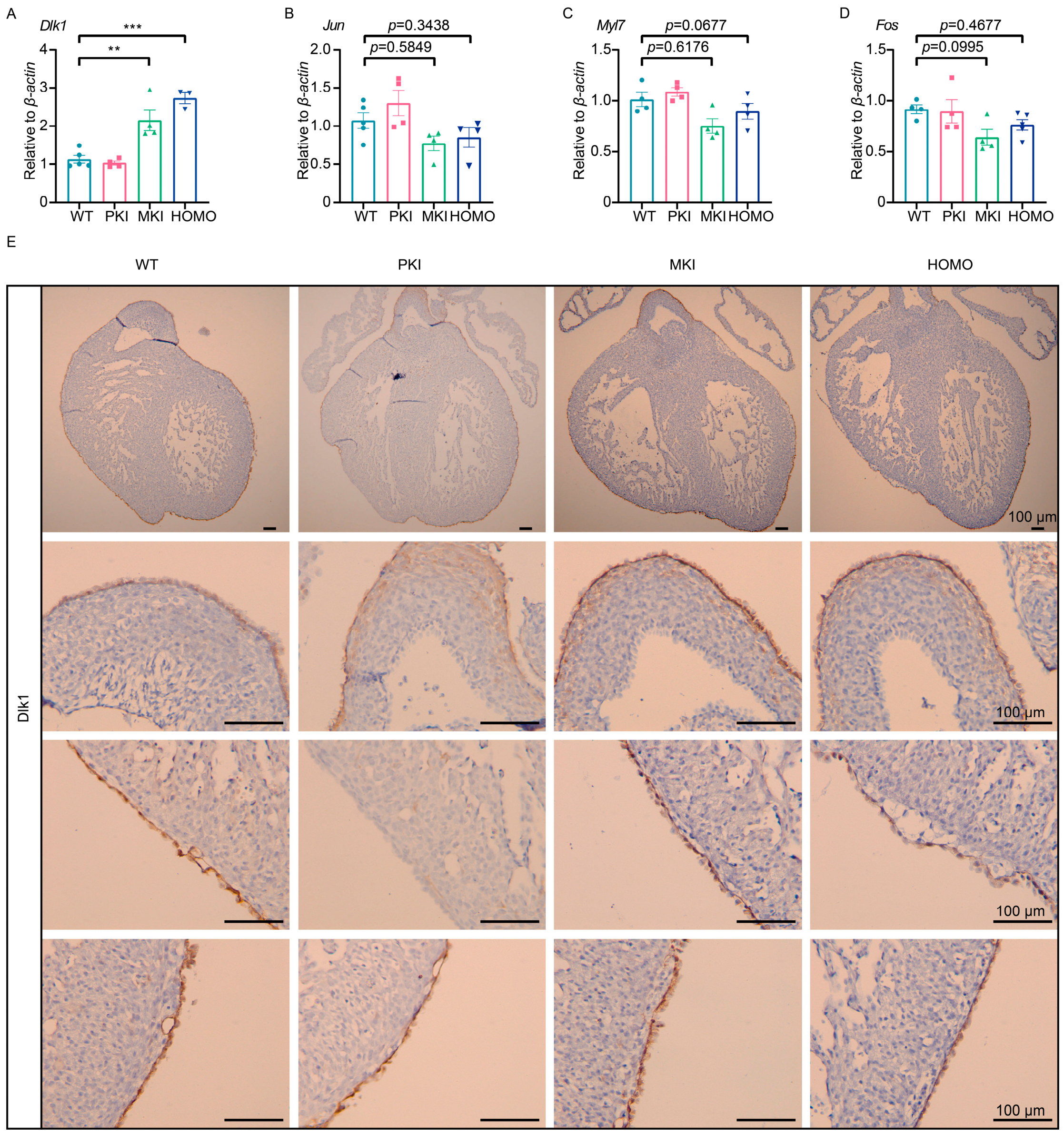

Figure S10 Graphical representations of the expression levels of *Dlk1*, *Jun*, *Myl7* and *Fos* in epicardial cells and immunohistochemistry showing Dlk1 expression in E14.5 embryonic hearts. (A-D) (*Dlk1* WT n=5, PKI, MKI n=4, HOMO n=3; *Jun* WT n=5, PKI, MKI, HOMO n=4; *Myl7* n=4; *Fos* WT, PKI, MKI n=4, HOMO n=5). (E) Immunohistochemistry showing Dlk1 expression in E14.5 embryonic hearts. Scale bar: 100 μm. The values represent the expression level relative to that of the *β-actin* expression level. Data are expressed as the mean ± SEM. ** $p < 0.01$; *** $p < 0.001$.

Table S1 Heart rates of surviving embryos at E12.5

Embryonic day	WT (bmp/20 s)	PKI (bmp/20 s)	MKI (bmp/20 s)	HOMO (bmp/20 s)
E12.5	25	19	25	28
	20	25	21	26
	19	23	18	23
	23	18	24	22
	21	22	20	27
	17	25	23	20
	29	23	21	22
	28	27		17
	26	18		23
	25			19
	24			
	23			
	23			

Table S2 Heart rates of surviving embryos at E14.5

Embryonic day	WT (bmp/20 s)	PKI (bmp/20 s)	MKI (bmp/20 s)	HOMO (bmp/20 s)
E14.5	22	27	23	28
	23	25	26	17
	23	26	25	21
	27	23	24	18
	28	20	20	15
	26	20	30	23
	26	19	14	15
	28	26	17	26
	29	23	18	21
	27	23	25	15
	30	28	22	15
	27	18	27	29
	27	31	19	25
	21	30	21	
	22	25	24	
	21	33		
	26	25		
	25	30		
	28	25		
	24	28		
	28			
	28			
	30			
	26			
	24			
	21			
	26			
	27			
	29			
	25			
	31			
	27			

Table S3 Heart rates of surviving embryos at E16.5

Embryonic day	WT (bmp/20 s)	PKI (bmp/20 s)
E16.5	27	28
	20	26
	25	27
	22	30
	21	27
	27	26
	26	29
	28	22
	20	20
	26	

Table S4 Primer sequences used for *in situ* hybridisation probe preparation

**Name**	**F(5'-3'）**	**R(5'-3'）**
Gtl2 probe	GGGAAATTGGAGGTGAGG	GGACAAGCGACAAAGAGG
Dlk1 probe	CCTCTTGCTCCTGCTGGCTTTC	GATGTGTTGCTCGGGCTGCTG
Rian probe	CAAATCTCCATGCACGGAAAG	GCCTTGACCATCATGAAGAC

Table S5 Primer sequences used for qRT-PCR

Name	F(5'-3')	R(5'-3')
β-actin	TACCACGGCATTGTGATGGACT	TTGATGTCACGCACGATTTCCCT
Dlk1	ACGGGAAATTCTGCGAAATA	CTTTCCAGAGAACCCAGGTG
Rian	TAGAGTCTCCCTTGAAAGTGG	TGGTATCTATAAGAACAGAGCTGA
Gtl2	CGAGGACTTCACGCACAAC	TTACAGTTGGAGGGTCCTGG
Mirg	GTTGTCTGTGATGAGTTCGC	CCTTGAACATCCGCTCC
Nppa	CTGCTTCGGGGGTAGGATTG	TAGATGAAGGCAGGAAGCCG
WT1	TCCGGTCAGCATCTGAAACC	ATGAGTCCTGGTGTGGGTCT
α-SMA	CGTTCAACCCTGAGACGCT	AGCGTCAGGATCCCTCTCTT
Fos	TACTACCATTCCCCAGCCGA	GCTGTCACCGTGGGGATAAA
Jun	TTTTCAAAGCTCGGCATCGC	GAGGGCATCGTCGTAGAAGG
Myl7	CTTCCTCACACTCTTCGGGG	AGGTGACCTCAGCCTGTCTA
U6	Forward Primer from Takara	Reverse Primer from Takara
miR-127	CTGAAGCTCAGAGGGCTCTGAT	mRQ 3' Primer from Takara
miR-154	TAGGTTATCCGTGTTGCCTTCG	mRQ 3' Primer from Takara
miR-409	AGGTTACCCGAGCAACTTTGCAT	mRQ 3' Primer from Takara
miR-495	GAAGTTGCCCATGTTATTTTTCG	mRQ 3' Primer from Takara
miR-300	TTGAAGAGAGGTTATCCTTTGT	mRQ 3' Primer from Takara
